# Discovery of glycerol phosphate and an immunogenic glycan motif in rhamnose-rich polysaccharides of *Streptococcus uberis*

**DOI:** 10.1186/s13567-025-01574-0

**Published:** 2025-07-07

**Authors:** Yao Shi, Göran Widmalm, Natalia Korotkova, Adrian Molenaar, Mark A. Holmes, Scott McDougall, Jetta J. E. Bijlsma, Nina M. van Sorge, Lindert Benedictus

**Affiliations:** 1 Department of Population Health Sciences, Division Farm Animal Health, Utrecht University, Utrecht, The Netherlands; 2 Department of Medical Microbiology and Infection Prevention, Amsterdam UMC Location University of Amsterdam, Amsterdam, The Netherlands; 3 Department of Chemistry, Arrhenius Laboratory, Stockholm University, Stockholm, Sweden; 4 Department of Molecular and Cellular Biochemistry, University of Kentucky, Lexington, USA; 5 Department of Microbiology, Immunology and Molecular Genetics, University of Kentucky, Lexington, USA; 6 AgResearch Ltd., Grassland Research Centre, Palmerston North, 4410 New Zealand; 7 Department of Veterinary Medicine, University of Cambridge, Cambridge, UK; 8 Cognosco, Anexa Veterinary Services, Morrinsville, 3300 New Zealand; 9 Discovery and Technology, MSD Animal Health, Boxmeer, The Netherlands; 10 Netherlands Reference Laboratory for Bacterial Meningitis (NRLBM), Amsterdam UMC Location AMC, Amsterdam, The Netherlands; 11 Present Address: Oncode Accelerator, Utrecht, The Netherlands

**Keywords:** Rhamnose-rich polysaccharides, glycerol phosphate, *Streptococcus uberis*, immunogenicity, bovine mastitis, bacterial cell wall, bacterial glycobiology, IgG

## Abstract

*Streptococcus uberis* is a causative pathogen of bovine mastitis with high genetic diversity. Rhamnose-rich polysaccharides (RPS) are abundant surface structures covalently anchored to peptidoglycan and represent promising vaccine candidates for several streptococcal pathogens. It was previously reported that the RPS of *S. uberis* strain 233 is composed of a repeating → 2)-α-l-Rha*p*-(1 → 3)-α-l-Rha*p*-(1 → disaccharide backbone decorated with α-d-Glc*p* side-chains. In this study, we identified a hitherto unknown glycerol phosphate (GroP) modification at the 6-OH of the Glc residue in *S. uberis* 233 RPS using nuclear magnetic resonance analysis. Comparative genomic analysis of 592 *S. uberis* genomes revealed significant diversity in the RPS biosynthesis gene cluster with six major RPS genotypes. RPS genotypes 1–4, representing 97.5% of the analyzed strains, all contained the rhamnan backbone biosynthesis genes shared between several streptococcal species, as well as a putative GroP transferase gene. Using rhamnan-reactive immune serum, we further demonstrated that rhamnan is a conserved and accessible glycan motif in *S. uberis* RPS genotype 1 and 2 strains, but this motif is inferred to be shielded by side-chains in genotype 4 strains. Importantly, experiments with sera from cattle, challenged intramammarily with *S. uberis*, revealed that the rhamnan backbone of *S. uberis* RPS is an immunogenic glycan motif and remained accessible to bovine IgG antibodies in the presence of single residue RPS side-chains. Overall, this study suggests that *S. uberis* RPS are modified with GroP and reports that RPS in most strains contain a conserved, immunogenic and antibody accessible rhamnan glycan motif.

**Supplementary Information:**

The online version contains supplementary material available at 10.1186/s13567-025-01574-0.

## Introduction

*Streptococcus uberis* (*S. uberis*) is an important bacterial pathogen primarily associated with mastitis in cattle, an inflammation of the udder that poses serious economic, health and welfare challenges in the dairy industry. *S. uberis* is a ubiquitous microorganism colonizing both the environment and animal body sites, such as the intestines, making it difficult to control exposure and infection [1–3]. Vaccination is a viable strategy for controlling *S. uberis* infection. The subunit vaccine UBAC® (HIPRA S. A.), which contains lipoteichoic acid from the biofilm adhesion component of *S. uberis*, reduced clinical signs and milk yield loss in a controlled immunization-challenge study [4]. Additionally, many experimental protein subunit vaccines, live vaccines, and inactivated vaccines have been tested over the past decades, but no commercial products have emerged [5, 6]. One specific challenge for the development of a broadly protective vaccine, is the high genetic variability of *S. uberis* [7, 8], comprising over 2000 sequence types (STs) as determined by multi-locus sequence typing (MLST), and multiple STs can circulate on a single farm [9, 10].

Capsular polysaccharides (CPS), which usually form the outermost layer of bacteria, have been successfully used as vaccine antigens [11]. However, *S. uberis* CPS consists of host-mimicking hyaluronic acid, making it unsuitable as a vaccine target. Secondary cell wall polysaccharides (SCWPs) are surface-exposed glycans of Gram-positive bacteria that are anchored to and protrude from the peptidoglycan layer and are abundantly expressed, making them attractive vaccine targets for opsonic antibodies [12]. For example, the group A carbohydrate (GAC) and group B carbohydrate (GBC) are conserved Lancefield antigens of *Streptococcus pyogenes* (*S. pyogenes*, also known as group A *Streptococcus*) and *Streptococcus agalactiae* (*S. agalactiae*, also known as group B *Streptococcus*), respectively, and have been identified as promising antigens for vaccine development [13, 14]. Additionally, wall teichoic acid (WTA) represents a potential vaccine candidate against (antibiotic-resistant) *Staphylococcus aureus* (*S. aureus*) [15].

*S. uberis* expresses two types of SCWPs: poly-glycerol phosphate WTA and rhamnose-rich polysaccharide (RPS) [16]. RPS of other streptococci typically contain a rhamnan backbone decorated with side-chains, and may account for up to 50% of the cell wall by weight [12, 17]. These glycans play crucial roles in bacterial homeostasis, growth, virulence, and cell division [12]. The genes involved in RPS biosynthesis are generally clustered on the streptococcal genome [12, 17]. The rhamnan backbone is synthesized by multiple glycosyltransferases and transported by an ABC transporter-dependent pathway, while the decoration of the rhamnan backbone with side-chains is mediated by multicomponent glycosylation systems [17–19]. The RPS structure of *S. uberis* strain 233 was identified as a [→ 2)-α-l-Rha*p*-(1 → 3)-α-l-Rha*p*-(1 →]_n_ backbone decorated with α-d-Glc*p* side-chains [16]. Interestingly, *Streptococcus mutans* (*S. mutans*) uses an identical side-chain decoration in its RPS, referred to as serotype c carbohydrate (SCC). However, in SCC, this side-chain is further modified with negatively-charged glycerol phosphate (GroP) by the GroP transferase SccH [20].

Beyond the reported chemical structure of the RPS of *S. uberis* strain 233 [16], no additional information is available on *S. uberis* RPS. The aim of this study is to identify the RPS biosynthesis gene cluster, characterize the genetic diversity within the *S. uberis* population and explore the immunogenicity of *S. uberis* RPS.

## Materials and methods

### Bacterial strains and culture conditions

*S. uberis* 233 is a non-encapsulated strain obtained from AgResearch, New Zealand [21]. *S. aureus* Newman Δ*spa*Δ*sbi* mutant does not bind IgG as a result of the deletion of protein A and Sbi [22] and was employed as a negative control. *S. mutans* serotype c strain Xc and its mutant Δ*sccN* (leading to a loss of the major Glc side-chain of SCC) were used as positive controls [23, 24]. All *S. uberis* [25], *S. aureus* and *S. mutans* strains used in this study are listed in Additional file 1. Bacteria were plated from −70 °C glycerol stock on either blood agar plate or Todd-Hewitt (Oxoid) agar supplemented with 0.5% yeast extract (Bacto) (THY plate). Multiple colonies were selected and grown overnight in THY broth at 37 °C with shaking at 120 rpm.

### Whole-genome sequencing and assembly

Genomic DNA of *S. uberis* 233 was isolated using the Maxwell® RSC Instrument (Promega) after overnight growth in THY broth. DNA was quantified by Qubit Fluorometer (Invitrogen). Library preparation and Illumina sequencing were performed by the Core Facility Genomics of Amsterdam UMC using the KAPA HTP Library Preparation Kit and MiSeq 150v3 (PE75) Kit, following the manufacturer’s instructions. Illumina reads were assembled as described previously [26].

### *Streptococcus uberis* genome collection and multi-locus sequence typing

All Biosample records in NCBI and genomes in PATRIC [27] with the keyword “*Streptococcus uberis*” were downloaded (final date: 26 May 2021). Sequence read archive data were assembled by SKESA (version 2.4.0) [28]. Genome qualities were assessed by QUAST (version 5.0.2) [29] and genomes were selected for further analysis based on the following criteria: (1) N50 higher than 10 kbp; (2) assembly with less than 200 contigs; (3) genome length between 1.5–3 Mbp; (4) GC content between 35.8%–36.8%. Metadata were collected from the online databases and corresponding publications. In total 592 *S. uberis* genomes were included in the final analysis (Additional file 2).

In silico MLST was performed using mlst (version 2.19.0) [30]. The MLST allele sequences and allele profiles were downloaded from PubMLST (date: 30 Aug 2022) [10, 31, 32].

### Identification of the *S. uberis* RPS biosynthesis gene cluster

The putative RPS biosynthesis gene clusters of *S. uberis* strains were identified by homologue searching using the Basic local alignment search tool (BLAST) [33]. Proteins were annotated by searching in UniProt [34] and InterPro [35]. Glycosyltransferases were also identified using CAZy [36], which is a database for carbohydrate-active enzymes. Genes were visualized using the packages gggenes (version 0.4.0) [37] and ggplot2 (version 3.3.2) [38] in R (version 4.0.3) [39, 40]. The alignments of the gene clusters were made by clinker (version 0.0.23) [41].

A custom-made database containing the nucleotide sequences of identified *S. uberis* RPS biosynthesis gene clusters was run in blastn (version 2.11.0) [33] against the *S. uberis* genome database using ABRicate (version 1.0.0) [42]. Genomes were classified as the same RPS genotypes based on a minimal coverage of 99% and identity of 97% of the nucleic acid sequences of the putative RPS biosynthesis gene cluster.

### Phylogenetic tree

Genome assemblies were annotated with Prokka (version 1.14.6) [43]. The core genome and pangenome were investigated by Panaroo (version 1.3.0) with built-in MAFFT to make a core genome alignment [44, 45]. A maximum likelihood phylogenetic tree was constructed by IQ-TREE (version 2.0.3) using a GTR + G model with 1000 bootstraps [46]. The number of constant sites in the core genome alignment were determined by SNP-sites (version 2.5.1) with the “-C” flag and fed to IQ-TREE using the “-fconst” flag [47]. The tree was visualized and annotated by iToL [48].

### Isolation and purification of RPS

Following overnight culture in THY broth*, S. uberis* 233 was diluted 1:20 in fresh THY broth and grown to late exponential phase (optical density around 0.7 at 600 nm). Bacteria were harvested by centrifugation at 4 °C and cell walls were isolated using the sodium dodecyl sulfate (SDS)-boiling procedure as previously described [23, 49, 50]. Cell walls were chemically *N*-acetylated and RPS was released by mild acid hydrolysis as outlined [50]. The RPS was purified by size-exclusion chromatography using BioGel P150 (Bio-Rad), equilibrated in sodium acetate-sodium chloride (NaOAc-NaCl) buffer, as described previously [50]. The concentration of rhamnose was measured by a modified anthrone assay [23, 50].

### Biotinylation of RPS

Purified RPS was biotinylated using biotin-amine (AxisPharm AP10507) through reductive amination. Briefly, the reaction was carried out in 80 mM NaOAc at pH 5, with a molar ratio of RPS to biotin to sodium cyanoborohydride (NaCNBH_3_) of 1:600:2500. After incubating overnight at room temperature in the dark, the biotinylated RPS was purified using 3 kDa MWCO filter units (Millipore) and washing with Milli-Q water and spinning repeated at least eight times.

### Phosphate assay

Purified RPS (corresponding to 700 nmol rhamnose) was incubated in 2 N hydrochloric acid (HCl) at 100 °C for 2 h, followed by neutralizing with sodium hydroxide (NaOH) in the presence of 62.5 mM HEPES pH 7.5 as verified using a pH indicator strip. Next, 50 μL of the acid hydrolyzed sample was incubated with 1 U alkaline phosphatase (ThermoFisher 78390), 10 μL 10 × alkaline phosphatase buffer and 1 μL 0.1 M magnesium chloride (MgCl_2_) at 37 °C overnight with rotation, in a total volume of 100 μL. Released phosphate was measured using a Malachite green phosphate assay kit (Cayman Chemical 10009325).

### Animal sera

Serum of a rabbit immunized with *S. mutans* serotype k carbohydrate (SKC) conjugate (RRHV3) was generously donated by Dr. Andrew D. Cox from the National Research Council in Canada [51]. RRHV3, hereafter referred to as anti-SKC serum, showed high antibody titers (≥ 1:16 000) against whole cells of *S. mutans* serotype c, f and k strains, in an ELISA assay [51].

Sera from cattle challenged twice intramammarily with *S. uberis* were obtained from a larger animal study performed from Oct 2013 to Jan 2014. All procedures and treatments were conducted according to European and Dutch law for animal experiments and welfare (approved by the national Dutch Animal Experiments Committee, No. MSU 13-033). Nine clinically healthy, recently calved and lactating Holstein-Friesians cows were infused intramammarily via the teat with 1 mL of *S. uberis* inoculum in peptone water per quarter in two quarters. General clinical assessment was performed, and udder health specific scoring was performed once daily at the morning milking (rectal temperature, milk somatic cell counts and udder and milk scores, Additional file 3). Animals reaching humane endpoints were treated with antibiotics and anti-inflammatory drugs or euthanized, depending on the clinical status. On day 14 after the first challenge, all cows received an antibiotic treatment. Cows that did not recover or developed non-*S. uberis*-challenge related disease were removed from the experiment. The second challenge was performed on day 34 after the first challenge in the same quarters according to the same procedure. Sera were collected on day 0 (pre-challenge) and day 42 (post-challenge) after the first challenge. Group A (*n* = 4) was challenged with 2.89 × 10^3^ CFU *S. uberis* strain bma (RPS genotype 1), followed by a heterologous challenge with 1.56 × 10^3^ CFU *S. uberis* strain FSL Z1-048 (RPS genotype 1). Group B (*n* = 5) was challenged with 5.23 × 10^2^ CFU *S. uberis* strain FSL Z1-048, followed by a homologous challenge with 1.56 × 10^3^ CFU.

Normal rabbit serum (Invitrogen) and fetal bovine serum (Gibco) were used as negative controls. All sera were heated at 56 °C for 30 min to inactivate complement before being used in assays.

### Bacteria antibody binding assay

Bacterial overnight cultures were diluted 1:10 in fresh THY and grown to exponential phase, corresponding to an optical density of 0.4 at 600 nm. Bacteria were harvested by centrifugation for 10 min at 3000 × *g*, 4 °C. The pellet was resuspended in 1 mL PBS supplemented with 0.1% bovine serum albumin (PBS-BSA). To stain the bacteria, bacterial suspension was mixed 1:1 with diluted serum in 96-well plates and incubated for 20 min at 4 °C. The final serum dilutions after mixing with bacteria were 1% for bovine serum and 0.5% for rabbit serum. The bacteria were washed with PBS-BSA, collected by centrifugation for 10 min at 3000 × *g*, 4 °C, and incubated with either (1) Protein G-Alexa Fluor 488 (Thermo Fisher P11065) at 1 μg/mL to test the total IgG or (2) Sheep anti-bovine IgM-FITC (Bethyl Laboratories A10-101F) at 5 μg/mL for 20 min at 4 °C in the dark. Following another washing with PBS-BSA, bacteria were fixed in 1% paraformaldehyde and analyzed by flow cytometry (BD FACSCanto II).

### Antibody binding assay using glycan-coated beads

Streptavidin beads (Invitrogen 11205D) were coated using either biotinylated *S. uberis* 233 RPS or synthetic biotinylated hexa-rhamnosides ([→ 2)-α-l-Rha-(1 → 3)-α-l-Rha-(1 →]_3_) as previously described [52]. 1 × 10^5^ beads in 12.5 μL were incubated with the same volume of diluted serum in a 96-well plate at 4 °C in PBS supplemented with 0.1% BSA and 0.05% Tween_20_ (PBS-BSA-T_20_ buffer) for 20 min with shaking. Serum concentrations were as described for the bacteria antibody binding assay. The beads were washed once using a plate magnet, then incubated with Protein G-Alexa Fluor 488 as described for the bacteria antibody binding assay for 20 min at 4 °C in the dark with shaking. After another wash using PBS-BSA-T_20_ buffer, the beads were resuspended in 100 μL PBS-BSA-T_20_ buffer and analyzed by flow cytometry (BD FACSCanto II).

### Antibody depletion assay

Bacterial overnight cultures were diluted 1:10 in fresh THY broth and grown to exponential phase, corresponding to an optical density of 0.4 at 600 nm. To deplete antibodies against bacterial surface structures, bacteria were harvested, resuspended in PBS and incubated with anti-SKC serum (RRHV3) in a 96-well plate at 4 °C for 30 min with shaking. The plate was centrifuged for 10 min at 3000 × *g*, 4 °C to pellet the bacteria. The supernatant (serum depleted of bacterial surface structures-reactive antibodies) was used to stain glycan-coated beads as described above.

To deplete anti-RPS antibodies from bovine serum, six serum samples with the highest anti-rhamnan IgG levels were selected. If a cow had high anti-rhamnan IgG levels in both pre- and post-challenge sera, the highest one was included, ensuring all the six samples were from different cows. Depletion was conducted by incubation of bovine sera with different amounts of purified 233 RPS at 4 °C for 15 min with shaking at 200 rpm; the (mock) depleted sera were used to stain glycan-coated beads immediately as described above.

### Nuclear magnetic resonance (NMR) spectroscopy

RPS isolated from *S. uberis* (1 or 4 mg, dry weight) was dissolved in D_2_O (0.55 mL). NMR experiments were conducted in 5 mm outer diameter NMR tubes on Bruker NMR spectrometers operating at ^1^H frequencies of 400 or 700 MHz at temperatures of 23 °C or 50 °C, respectively, using experiments suitable for resonance assignments of glycans [53, 54]. ^1^H NMR chemical shifts were referenced to internal sodium 3-trimethylsilyl-(2,2,3,3-^2^H_4_)-propanoate (*δ*_H_ 0.0), ^13^C chemical shifts were referenced to external dioxane in D_2_O (*δ*_C_ 67.4) and ^31^P chemical shifts were referenced to external 2% H_3_PO_4_ in D_2_O (*δ*_P_ 0.0). Acquired NMR data were processed and analyzed using the TopSpin® software from Bruker.

### Data analysis

Data acquired by flow cytometry were analyzed using FlowJo (version 10.10.0). The gating strategies are shown in Additional file 4. The populations of single bacteria or single beads were gated, and the geometric mean fluorescence intensity (GMFI) of the Alexa Fluor 488 was extracted. The GMFI of background signal was subtracted to calculate the ΔGMFI, which was used to reflect specific antibody binding. For antibody binding to bacteria, bacteria stained with secondary antibody or protein G only were used as background signal. For beads assays using rabbit serum, glycan coated beads stained by protein G only were used as background signal. Because bovine antibodies can bind to uncoated beads, for bead assays using bovine serum, uncoated beads stained with both bovine serum and protein G were used as background signal. Statistics and visualizations were done using GraphPad Prism (version 10.2.0). *p*-values are shown as * *p* ≤ 0.05; ** *p* ≤ 0.01; *** *p* ≤ 0.001; **** *p* ≤ 0.0001; ns, not significant (*p* > 0.05). The specific statistical tests are indicated where *p*-values are reported.

## Results

### Identification of the RPS biosynthesis gene cluster of *S. uberis* 233

**Figure 1:**
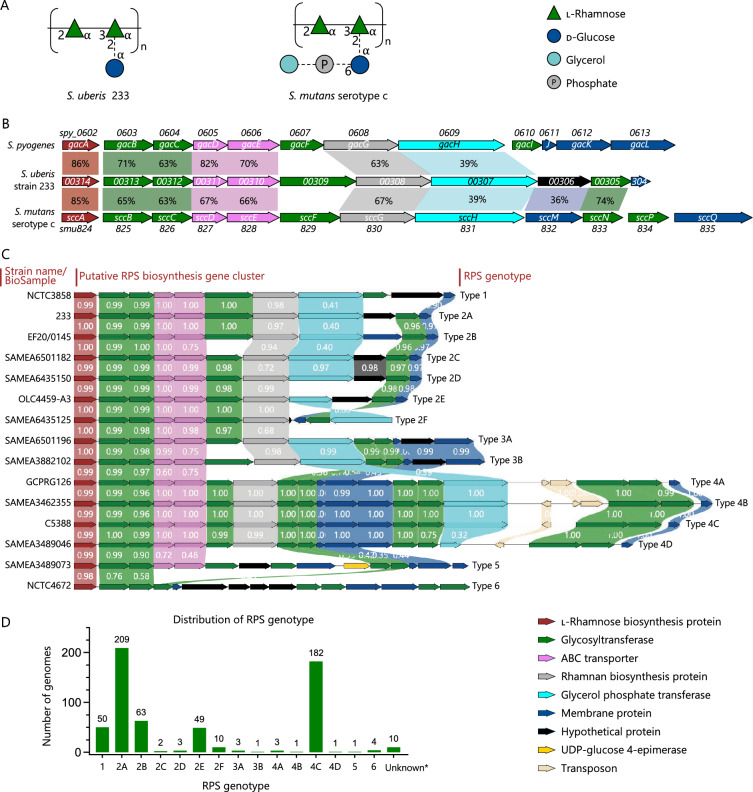
**Bioinformatic analysis of the RPS biosynthesis gene clusters in *S. uberis*. A** Published structure of the repeating units of the *S. uberis* 233 RPS [16] and the *S. mutans* serotype c carbohydrate (SCC). SCC is decorated with the major Glc side-chain [55]. The dashed lines denote partial substitution by the side-chain or glycerol phosphate entities. **B** A comparison of the identified RPS biosynthesis gene cluster in *S. uberis* strain 233, the group A carbohydrate (GAC) biosynthesis gene cluster of *S. pyogenes* strain MGAS5005 (NCBI accession: NC_007297.2), and the SCC biosynthesis gene cluster of *S. mutans* strain UA159 (NCBI accession: NC_004350.2). The percentage of amino acid identity greater than 30% between the homologous proteins are indicated. **C** Alignment of the *S. uberis* RPS biosynthesis gene clusters grouped into six main genotypes based on the analysis of 592 *S. uberis* genome sequences. Genomes were defined as the same RPS genotypes based on a minimal coverage of 99% and identity of 97% of the whole cluster at nucleotide level. Arrows are drawn to scale according to gene size, with each function indicated by color. Regions with greater than 30% amino acid identity are highlighted. **D** Distribution of *S. uberis* RPS genotypes within the *S. uberis* genome dataset (*n* = 592). * The RPS gene clusters of these genomes were not located in a single contig.

While the glycan structure of the RPS of *S. uberis* 233 has been reported [16], the biosynthesis gene cluster has not been identified. Since the glycan structure of *S. uberis* 233 RPS is identical to the *S. mutans* SCC, which is decorated with Glc as major side-chain [55] (Figure 1A), the *S. uberis* 233 genome was searched for homologues of the SCC biosynthesis genes. Based on homology and predicted gene function, a putative 11-gene cluster was identified (Figure 1B). The cluster starts with gene *00314*, encoding a putative dTDP-4-dehydrorhamnose reductase (RmlD) which catalyzes the final step of dTDP-l-rhamnose biosynthesis, followed by genes for glycosyltransferases (including a rhamnan biosynthesis protein), ABC transporter subunits, a GroP transferase, a membrane protein and a hypothetical protein. The genetic structure (function and order of genes) of the RPS biosynthesis gene cluster of *S. uberis* 233 is highly similar to that of *S. pyogenes*, *Streptococcus dysgalactiae* subspecies *equisimilis* (*S. dysgalactiae* subsp. *equisimilis*) and *Streptococcus equi* (*S. equi*), as well as *S. mutans* [12]. In *S. pyogenes* and *S. mutans*, the functions of some enzymes, encoded by the RPS biosynthesis clusters, have been elucidated and confirmed experimentally [13, 18–20, 23]. Based on homology and predicted function, the first seven genes (*00314*–*00308*) of the *S. uberis* 233 RPS gene cluster are predicted to encode proteins that synthesize and transport the rhamnan backbone across the cytoplasmic membrane: glycosyltransferases encoded by genes *00313*, *00312*, *00309* and *00308* are predicted to perform the initiation, elongation and capping of the rhamnan backbone, whereas the two ABC transporter subunits encoded by genes *00311* and *00310* flip the rhamnan backbone from the cytosol to the outer leaflet of the cytoplasmic membrane. Gene *00305* encodes a putative GT-A fold glycosyltransferase with 74% amino acid identity to *S. mutans* SccN, which synthesizes β-Glc-phosphate-undecaprenyl, the glycolipid required for modification of SCC with α-d-Glc [55] (Figure 1B). Gene *00307* is predicted to encode a GroP transferase, which in other streptococci catalyzes the transfer of GroP onto side-chains [20, 23]. The other two genes, *00304* and *00306*, are likely implicated in the formation and attachment of the Glc side-chain.

### Genetic diversity in the RPS biosynthesis gene cluster within the *S. uberis* population

RPS show a different level of structural variation in different species. For example, *S. pyogenes* GAC is conserved in all *S. pyogenes* strains [13], whereas four serotype-defining specific RPS variants have been identified in *S. mutans* strains [51, 55]. Genetic variation in the biosynthesis gene cluster provides an indication on RPS structural variation. To gain insight whether the published *S. uberis* RPS structure is representative for the *S. uberis* population, we analyzed the genetic variation in the *S. uberis* RPS biosynthesis gene cluster using 592 *S. uberis* genomes collected from public databases. Fifteen RPS gene cluster genotypes were identified and grouped into six main genotypes based on the genetic structure of the cluster and homologies of the putative side-chain-related glycosyltransferases (Figure 1C). Overall, the *S. uberis* RPS genotypes 1–4 represented 97.5% of the analyzed genomes and these strains all contained the set of canonical genes that are required for the rhamnan backbone biosynthesis [19] and the putative GroP modification. Genotypes 5 and 6, representing 0.84% of the strains, did not contain this core set of genes. The distribution of RPS genotype is shown in Figure 1D.

Strains within RPS genotypes 1 and 2 had a single putative side-chain-related glycosyltransferase, but they were not homologous (less than 30% identity at protein level). Based on homology in the putative side-chain glycosyltransferase, genotype 2 strains were grouped together, and subdivided into types 2A to 2F based on sequence variation across the entire RPS gene cluster. The RPS genotype 3 contained two putative side-chain-associated glycosyltransferases, but none of them were homologous with RPS genotypes 1 and 2. Genotype 4 subtypes contained multiple glycosyltransferases predicted to be involved in side-chain biosynthesis, suggesting a complex side-chain structure. Alternatively, the glycosyltransferases could also be involved in modifications of the rhamnose backbone, similar to *S. agalactiae* [12, 56]. Interestingly, genotype 4 subtypes included transposons, suggesting increased genetic plasticity for this region [57, 58].

**Figure 2:**
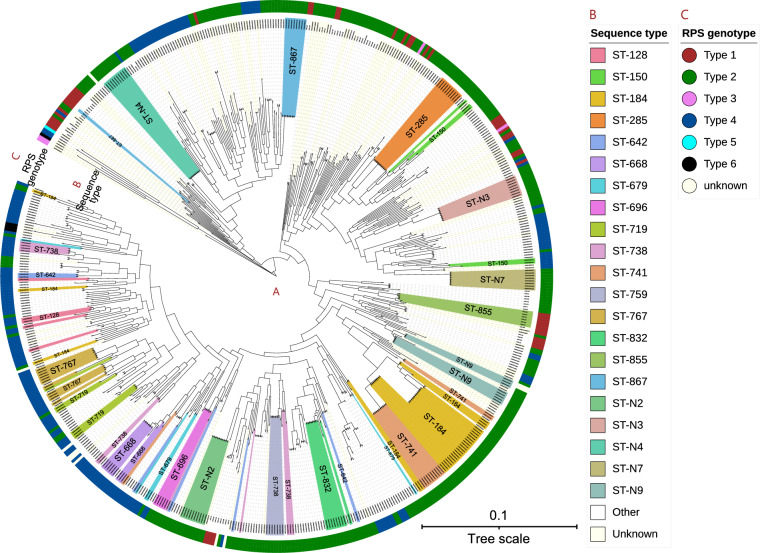
**Genetic variation of the *S. uberis* RPS biosynthesis gene clusters at population level.** **A** Core-genome maximum-likelihood phylogeny based on the collected 592 *S. uberis* genomes reconstructed using IQ-TREE. **B** Multi-locus sequence type of the strains. Frequently occurring sequence types (STs) are indicated with colors. New allele profiles that were not presented in the PubMLST database (date: 30 Aug 2022) were identified as ST-Nx. **C** RPS genotype of the strains.

The RPS genotypes were not associated with *S. uberis* MLST or core genome phylogeny (Figure 2), which indicates that the RPS biosynthesis gene cluster, especially the putative genes encoding the side-chain biosynthesis, can be transferred horizontally between different *S. uberis* STs, similar to the CPS of *Streptococcus pneumoniae* and *Streptococcus suis* [59, 60].

### *S. uberis* 233 RPS is modified with glycerol phosphate

All of the RPS genotypes that contained the core genes predicted to synthesize the rhamnan backbone (types 1–4) also contained a GroP transferase gene. The GroP modification of *S. pyogenes* GAC and *S. mutans* SCC has been attributed to the GroP transferase gene located in GAC and SCC biosynthesis gene clusters [20]. Although a GroP modification was not identified in the published structure of *S. uberis* 233 [16], the presence of the putative GroP transferase 00307 implies *S. uberis* 233 RPS is similarly modified with GroP (Figure 1B). To verify this hypothesis, purified RPS was acid-hydrolyzed and subjected to alkaline phosphatase to release glycerol and phosphate. Phosphate was subsequently quantified by malachite green assay. The results showed that phosphate was present in the *S. uberis* 233 RPS, with a ratio of 2.2 phosphate molecules to 50 rhamnose residues (Additional file 5). This result strongly suggests that GroP is part of *S. uberis* 233 RPS.

**Figure 3:**
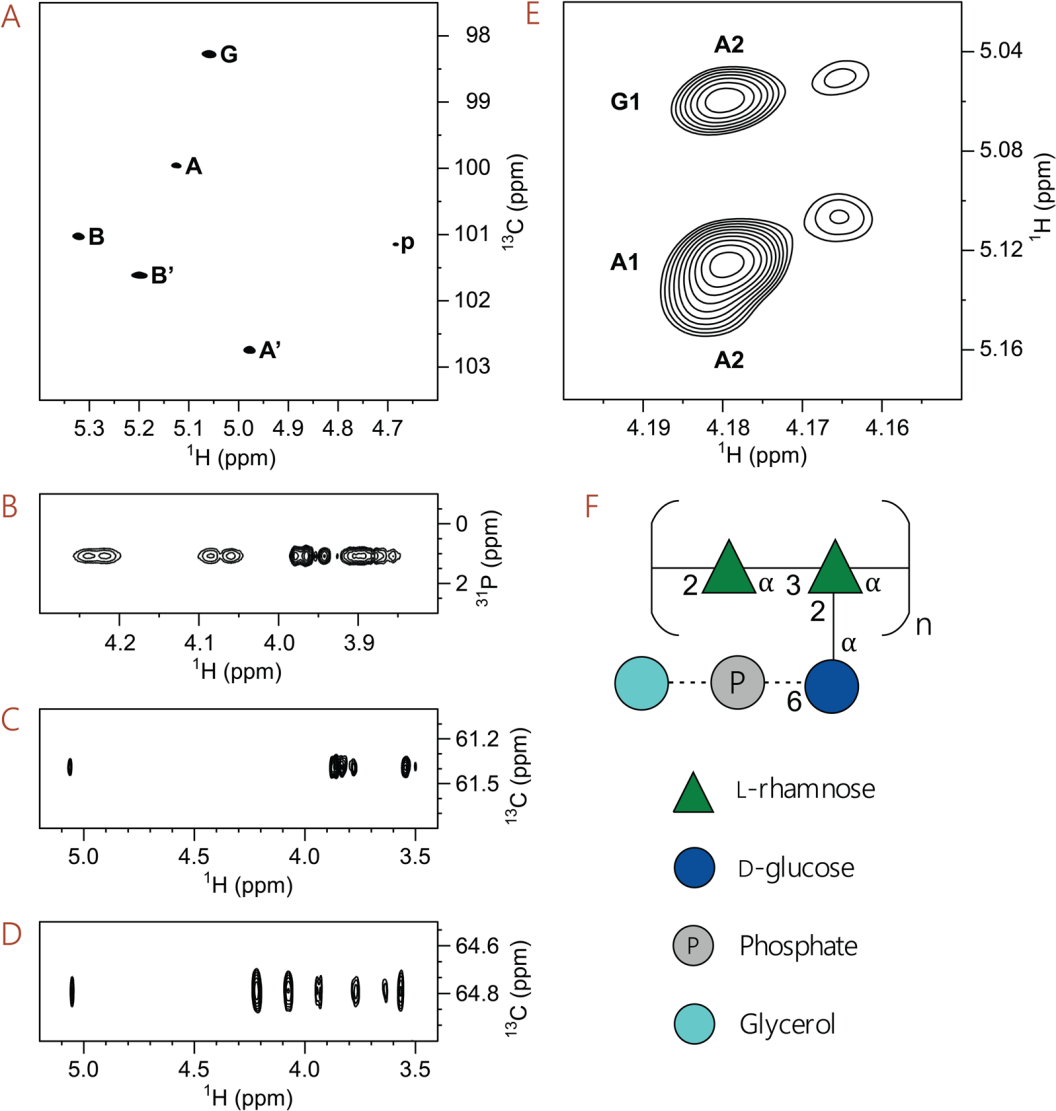
**NMR spectral regions of the *S. uberis* 233 RPS and a schematic representation of partially phosphoglycerol-substituted trisaccharide repeating units (RUs).** **A** Anomeric region of a ^1^H,^13^C-HSQC NMR spectrum in which the cross-peaks from the sugar residues of the glucosyl-containing trisaccharide RUs are labelled by **A**, **B** and **G** (cf. Table 1), the linear rhamnan backbone residues are labelled by **A’** and **B’**, and a tentatively assigned sugar residue being part of the primer region of the polysaccharide is labelled by **p**. **B** ^1^H,^31^P-HMBC NMR spectrum of the RPS identifying phosphodiester-linked entities. **C** Spectral region from a ^1^H,^13^C-HSQC-TOCSY NMR experiment (*τ*_mix_ 200 ms) showing correlations from C6 to protons in glucosyl residue **G**. **D** Spectral region from a ^1^H,^13^C-HSQC-TOCSY NMR experiment (*τ*_mix_ 200 ms) showing correlations from C6 to the seven-proton glucosyl spin-system of the α-d-Glc*p*6*P*(*S*)Gro-(1 → 2)-linked side-chain residue in the RPS. **E** Spectral region from a ^1^H,^1^H-NOESY NMR experiment at 700 MHz (*τ*_mix_ 200 ms) depicting cross-peaks from anomeric protons (**G1** at 5.06 ppm and **A1** at 5.12 ppm) in the trisaccharide repeating unit of the RPS to H2 at ~4.18 ppm (**A2**) of the branched rhamnosyl residue and the corresponding ones, at 5.05 ppm and at 5.11 ppm, respectively, of lower intensity at ~4.165 ppm in which the RU contains an α-d-Glc*p*6*P*(*S*)Gro-(1 → 2)-linked side-chain residue. **F** Canonical structure of the trisaccharide RU, where the dashed lines denote partial substitution by a GroP moiety.

To confirm the presence of GroP and to establish the location of the GroP modification on *S. uberis* 233 RPS, purified RPS was analyzed by NMR spectroscopy. The 2D ^1^H,^13^C-HSQC NMR spectrum of *S. uberis* RPS showed cross-peaks in the anomeric region originating from the rhamnan backbone repeating unit (RU) and the branched RU containing a glucosyl residue as a side-chain (Figure 3A), which are consistent with the published NMR spectrum of *S. uberis* RPS [16]. Furthermore, based on the magnitude of ^1^*J*_C1,H1_ in the range of 171–174 Hz, the anomeric configuration of these sugar residues is α. In the ^1^H,^1^H-NOESY NMR spectrum, cross-peaks were observed, inter alia, between anomeric protons of residues **B**/**B’** and H3 of residues **A**/**A’**, respectively, between H1 of **B**/**B’** and H5 of residues **A + 1**/**A’ + 1** (which are comparable to interresidue ^1^H,^1^H-NOEs for a corresponding rhamnose-containing disaccharide [61]) as well as between anomeric protons in residues **A** and **G**. Taken together these results are consistent with the reported structures of the RPS isolated from *S. uberis* 233 [16] and those present in the RPS from *S. mutans* SCC [51, 62]. In the 2D spectrum, a ^1^H,^13^C-correlated cross-peak of low intensity (**p**) was observed at *δ*_H_/*δ*_C_ 4.685/101.1 (Figure 3A), ^1^*J*_C1,H1_ = 160 Hz, and in a ^1^H,^13^C-HMBC NMR spectrum a conspicuous correlation was observed at *δ*_H_/*δ*_C_ 4.685/71.3 consistent with a large ^2^*J*_C2,H1_ coupling constant [63, 64]. These correlations are tentatively assigned to originate from a β-l-Rha*p* sugar residue being part of the primer region of the polysaccharide.

The ^31^P NMR spectrum revealed a resonance at *δ*_P_ 1.1 and a ^1^H,^31^P-HMBC NMR spectrum of the RPS showed cross-peaks to protons at *δ*_H_ 4.23, 4.07 and ~4.00–3.85 (Figure 3B). In the ^1^H,^13^C-HSQC NMR spectrum, correlations were observed, inter alia, *δ*_H_/*δ*_C_ 3.92/71.6 and *δ*_H_/*δ*_C_ 3.96,3.89/67.3, and in the 1D ^13^C NMR spectrum it was evident that the former methine carbon was spin–spin coupled with 7.5 Hz. The latter methylene carbon was spin–spin coupled with 5.7 Hz, consistent with a phosphodiester-linked glycerol substituent [20, 55]. To investigate the structure in greater detail the ^1^H and ^13^C NMR chemical shifts of the branched RU were first predicted using the CASPER program (Additional file 6A) [65, 66] and subsequently assigned (Table 1). This analysis included all transglycosidic correlations in the ^1^H,^13^C-HMBC NMR spectrum defining the structure and a comparison (Additional file 6B) revealed that predicted and experimental data were in excellent agreement with each other. ^1^H,^13^C-HSQC-TOCSY NMR spectra (Figures 3C and D) and chemical shift predictions (Additional file 6C), using again the CASPER program, revealed that the ^1^H,^1^H spin-systems of the glucosyl side-chain residue differed significantly. Experimental ^13^C NMR spectra showed a chemical shift displacement of C6 from 61.4 to 64.8 ppm upon phosphorylation at O6, and that H6 protons reside at < 3.9 ppm in the glucosyl residue but at > 4.0 ppm when substituted by a phosphodiester group (Figures 3B–D). Notably, whereas the anomeric proton of the non-substituted glucosyl group has *δ*_H1_ 5.06 (Figure 3C and Table 1) that of the glucosyl residue substituted at position 6 has *δ*_H1_ 5.05 (Figure 3D) and additional chemical shifts at *δ*_H_ 4.22, 4.07, 3.94, 3.77, 3.64 and 3.57 of the spin-system contained seven protons. The GroP-substituted residue is linked to the branched rhamnosyl residue like in the branched RU containing the non-substituted glucosyl residue. This was confirmed by a ^1^H,^1^H-NOESY NMR experiment in which correlations in the 2D NMR spectrum were observed for the anomeric glucosyl residues at *δ*_H1_ 5.06 and *δ*_H1_ 5.05 to H2 of the rhamnosyl residues at *δ*_H2_ 4.18 and *δ*_H2_ 4.165, respectively (Figure 3E). The branched RU of the *S. uberis* RPS is partially substituted by a phosphoglycerol group at O6 (Figure 3F), like in *S. mutans* RPS SCC [55] and the absolute configuration of the substituent is assumed to be *sn*-Gro-1-*P* as found in *S. pyogenes* GAC [20].

**Table 1:** ^**1**^**H and **^**13**^**C NMR chemical shifts (ppm) of a branched RPS from *S. uberis* in D**_**2**_**O at 323 K referenced to TSP (*δ***_**H**_** 0.00) and dioxane in D**_**2**_**O (*δ***_**C**_** 67.40)**

Residue	Label	1	2	3	4	5	6	6
→ 2,3)-α-l-Rha*p*-(1 → 2)	**A**	5.12	4.18	4.02	3.72	3.78	1.30	
		100.0	76.6	75.4	73.2	70.6	17.3	
→ 2)-α-l-Rha*p*-(1 → 3)	**B**	5.32	4.08	3.87	3.51	3.80	1.35	
		101.0	79.0	70.8	73.2	69.9	17.7	
α-d-Glc*p*-(1 → 2)	**G**	5.06	3.55	3.78	3.50	3.87	3.82	3.87
		98.3	72.2	73.4	70.3	73.0	61.4	

### Rhamnan is a conserved glycan motif of RPS genotype 1 and 2 strains

**Figure 4:**
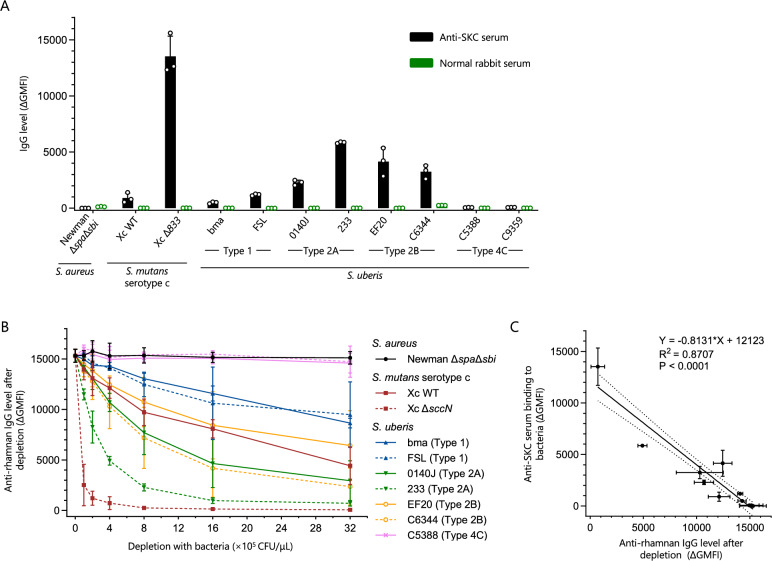
**An antigenic rhamnan motif is shared by the RPS expressed by *S. uberis* genotype 1 and 2 strains. A** Binding of IgG from anti-SKC or control rabbit serum to different bacterial strains measured by flow cytometry. Anti-SKC serum was generated by immunizing a rabbit with a *S. mutans* serotype k carbohydrate (SKC) conjugate [51]. *S. aureus* Newman Δ*spa*Δ*sbi* was employed as a negative strain control. *S. mutans* Xc Δ*sccN,* deficient in the major Glc side-chain of SCC [55], was used as a positive strain control. The schematic structures of the RPS variant from *S. mutans* Δ*sccN* and SKC are shown in Additional file 7A. Data from three separate bacterial inoculations are presented as mean values ± SD. **B** Anti-rhamnan IgG reactivity in anti-SKC serum before and after depletion by different *S. uberis* RPS genotype strains. Live bacteria were incubated with anti-SKC serum to deplete the antibodies against bacterial surface structures. The original (undepleted) and depleted serum were analyzed for anti-rhamnan reactivity using synthetic rhamnan coated beads and flow cytometry. Data from three separate bacterial inoculations are presented as mean values ± SD. **C** Correlation between the binding of anti-SKC serum to bacteria (Y-axis) and the respective anti-rhamnan IgG level after depletion by different bacteria at 8 × 10^5^ CFU/μL (X-axis).

The RPS of *S. uberis* 233, *S. mutans* SCC and *S. pyogenes* GAC share the same rhamnan backbone RU ([→ 2)-α-l-Rha*p*-(1 → 3)-α-l-Rha*p*-(1 →]_n_) [12, 16, 17]. Together with *S. uberis* strains of RPS genotypes 1–4, they all show a high degree of homology in the first seven RPS genes that are predicted to encode the machinery to synthesize the rhamnan backbone (Figure 1). To provide evidence that the RPS of all genotype 1–4 strains contain a rhamnan structure, we stained *S. uberis* strains of diverse RPS genotypes and control strains with anti-SKC rabbit serum [51] and control serum from a non-immunized rabbit and subsequently measured antibody (IgG) binding of whole bacteria using flow cytometry (Figure 4A). The anti-SKC serum stained a *S. mutans* serotype c wildtype (WT) strain, which expresses a heterologous RPS (Additional file 7A), but did not bind to *S. aureus*, which expresses WTA instead of RPS (Figure 4A). The anti-SKC serum recognized synthetic rhamnan (Figure 4B) and, compared to the wildtype *S. mutans* serotype c strain, demonstrated increased staining of *S. mutans* Δ*sccN* that expresses a RPS rhamnan backbone with only the minor Glc side-chains [55] (Figure 4A and Additional file 7A). Together this indicates that the anti-SKC serum contains IgG specific for the rhamnan backbone, which are partially blocked from binding by the presence of side-chains. Antibodies from the anti-SKC serum also recognized *S. uberis* 233 and purified *S. uberis* 233 RPS (Additional file 7B). Since the RPS of *S. uberis* 233 and *S. mutans* SCC are structurally identical, this strongly suggests binding of rhamnan-specific IgG to the RPS backbone of *S. uberis* 233. Interestingly, anti-SKC serum also reacted with *S. uberis* strains expressing RPS genotype 1 and 2, binding of rhamnan-specific IgG to other *S. uberis* strains is in line with our hypothesis that all strains with the canonical rhamnan synthesis genes express a rhamnan backbone. In contrast, two strains possessing the RPS genotype 4C were not recognized by the anti-SKC serum (Figure 4A). We hypothesized that glycosyltransferases of the RPS gene cluster type 4 catalyze the synthesis of more complex side-chains that shield the rhamnan backbone from antibody recognition.

To confirm that the reactivity of anti-SKC serum to *S. uberis* was conferred by the anti-rhamnan antibodies, rhamnan specific IgG levels in anti-SKC serum were measured before and after depletion of *S. uberis*-reactive antibodies (Figure 4B). The anti-SKC serum was incubated with whole *S. uberis* bacteria to deplete antibodies against bacterial surface structures and the original (undepleted) and depleted sera were used to stain synthetic rhamnan coated beads and subsequently measure the anti-rhamnan IgG levels using flow cytometry. All strains possessing the RPS genotype 1 and 2 depleted rhamnan-specific IgG from anti-SKC serum, demonstrating that the rhamnan backbone in these strains is accessible to antibodies. The variable effectiveness of depletion is likely the result of partial shielding of the rhamnan backbone by side-chains. Consistently, the lack of anti-rhamnan antibody depletion by the RPS genotype 4C strains and the efficient depletion by the *S. mutans* Δ*sccN* mutant correlates with the presence of complex and minor side-chains, respectively (Figure 4B).

There was a strong correlation (R^2^ = 0.87, *p* < 0.0001) between anti-SKC serum staining of the strains and anti-rhamnan IgG depletion by the respective strains (Figure 4C), indicating that the depletion was specific. No correlation was observed between staining with a control serum and depletion of anti-rhamnan IgG (Additional file 7C). In a similar depletion assay, depletion of anti-SKC serum by *S. mutans* Δ*sccN* and heterologous genotype 1 and 2 RPS strains reduced binding to *S. uberis* 233 RPS-coated beads, confirming the presence of shared antibody epitopes within the RPS structures between these respective strains (Additional file 7B).

### The conserved rhamnan backbone is an immunogenic glycan motif in cattle

**Figure 5:**
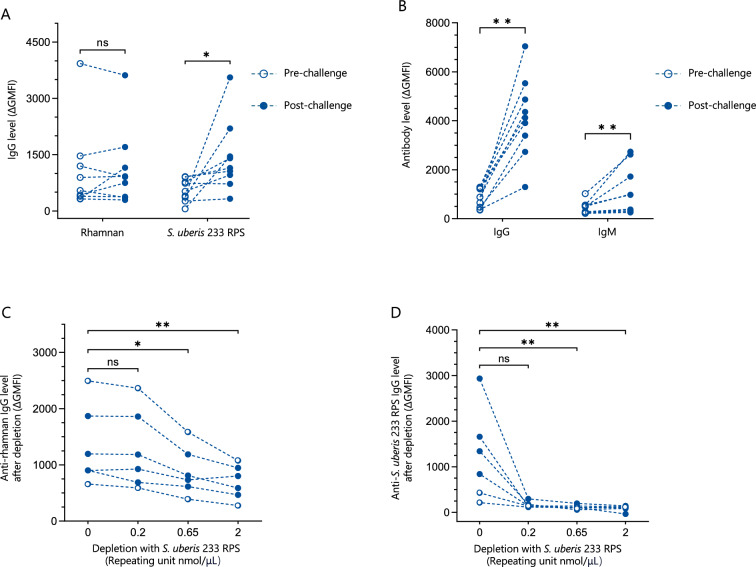
**The rhamnan backbone of *S. uberis* RPS is an immunogenic glycan motif in cattle. A** Serum IgG levels against synthetic rhamnan or purified *S. uberis* 233 RPS in cattle (*n* = 9) pre- and post-intramammary challenge with *S. uberis*. Streptavidin beads were coated with biotinylated antigen (synthetic rhamnan or *S. uberis* 233 RPS) and stained with serum. **B** Antibody levels against *S. uberis* whole cells in sera of cattle (*n* = 9) pre- and post-intramammary challenge with *S. uberis*. Live bacteria were incubated with bovine sera to stain bacterial surface structures. Antibody binding was assessed by flow cytometry. Pre- and post-challenge sera were compared using Wilcoxon matched pairs signed rank test followed by Holm-Šídák’s multiple comparisons test. **C** and **D** Anti-rhamnan IgG reactivity (**C**) and anti-233 RPS IgG reactivity (**D**) in bovine sera (*n* = 6 animals, two pre-challenge (open circle) and four post-challenge (filled circle)) before and after depletion by *S. uberis* 233 RPS. Purified *S. uberis* 233 RPS was incubated with bovine sera to deplete the antibodies against the antigenic epitopes on *S. uberis* 233 RPS. The depleted and mock-depleted sera were analyzed for anti-rhamnan IgG reactivity using rhamnan-coated beads (**C**) and anti-233 RPS IgG reactivity using 233 RPS-coated beads (**D**). Antibody level was measured by flow cytometry. Depletion was assessed by Friedman test followed by Dunn’s multiple comparisons test.

We next explored whether rhamnan is immunogenic in cattle and whether the rhamnan backbone of RPS is accessible by bovine antibodies. Sera were obtained from healthy lactating cows without mastitis, that are naturally exposed to diverse streptococci that are part of the normal microbiome [67, 68]. Before *S. uberis* challenge the sera contained variable IgG levels against synthetic rhamnan-coated beads and to purified *S. uberis* 233 RPS (Figure 5A). Two intramammary challenges with *S. uberis* induced a strong serum IgG and IgM antibody response against whole *S. uberis* bacteria (Figure 5B), but did not increase the rhamnan specific serum IgG levels (Figure 5A). In contrast, IgG reactive with purified *S. uberis* 233 RPS increased significantly after intramammary challenge with *S. uberis* strains expressing genotype 1 RPS (Figure 5A). Taken together, these data indicate that the epitopes on rhamnan are accessible by bovine antibodies, and that *S. uberis* challenge boosted antibody responses against native *S. uberis* RPS, but not to the rhamnan backbone.

To confirm that rhamnan-specific bovine IgG can recognize *S. uberis* RPS, cattle sera were depleted using increasing amounts of purified *S. uberis* 233 RPS. Subsequently, mock-depleted and 233 RPS depleted bovine sera were used to stain synthetic rhamnan-coated beads (Figure 5C) and 233 RPS coated beads (Figure 5D). The results revealed that purified native *S. uberis* 233 RPS can deplete rhamnan-specific IgG in bovine sera (average depletion of 46% at 2 nmol 233 RPS RU/μL), confirming that the glucose side-chains on the *S. uberis* 233 RPS do not prevent binding of the rhamnan specific cattle IgG to the rhamnan backbone. Whereas rhamnan-specific IgG was partially depleted, 233 RPS-specific IgG were almost completely depleted (average depletion of 84% at 2 nmol 233 RPS RU/μL).

## Discussion

RPS are abundant surface glycans anchored to the peptidoglycan layer of the streptococcal cell wall, and are prospective candidates for glycoconjugate vaccines [12–14, 51, 69]. Whereas GAC is highly conserved across *S. pyogenes* strains [13], four known serotype-specific RPS variants are produced by *S. mutans* strains [51]*.* Here we identified the putative biosynthesis gene clusters of *S. uberis* RPS and reported the presence of a GroP modification on the RPS in *S. uberis* strain 233. Although the RPS biosynthesis gene clusters are highly variable across the *S. uberis* population, over 97.5% of the analyzed strains share a set of canonical genes putatively involved in the synthesis of the RPS rhamnan backbone. Based on antibody reactivity and specific depletion methods, we demonstrated that the RPS expressed by *S. uberis* genotype 1 and 2 strains has a rhamnan backbone. We further demonstrated that the rhamnan backbone presents an immunogenic glycan motif in cattle. Antibody recognition of the rhamnan motif is influenced by side-chain decorations of RPS. While the rhamnan backbone remains accessible to bovine IgG in the presence of simple side-chains, it is likely to be shielded by the presence of complex side-chains.

Both *S. pyogenes* GAC and *S. mutans* SCC are modified with GroP, which provides a negative charge to the polysaccharides and bacterial cell surface as a whole [20, 50, 55]. The GroP moieties on GAC and SCC were overlooked in earlier studies [20] and have not been identified in a previous characterization of the 233 RPS glycan structure [16]. In this study, we unambiguously confirmed the presence and location of a GroP modification on *S. uberis* strain 233, both by phosphate analysis and NMR spectroscopy. Bioinformatic analysis of the *S. uberis* biosynthesis clusters showed that all RPS genotypes, except for the rare genotypes 5 and 6, have a putative GroP transferase, suggesting that *S. uberis* RPS variants are generally modified with GroP. In line with other streptococci, where the GroP modifications increase susceptibility to cationic antimicrobial proteins such as lysozyme, human group IIA secreted phospholipase A_2_ (hGIIA) and human histones [20, 23, 70], the GroP moieties on the *S. uberis* RPS are likely important for homeostasis and pathogenicity. It is unknown whether GroP modification affects the immunogenicity of RPS. In addition, we confirmed the main chemical structure of *S. uberis* 233 RPS using NMR spectroscopy. However, the NMR spectra and glycosyl linkage analysis using gas chromatography-mass spectrometry (GC–MS) of the partially methylated alditol acetate (PMAA) derivatives (data not shown) indicated that additional structural elements of low abundance may be present in the RPS material.

Although the RPS biosynthesis gene clusters within the *S. uberis* population have significant diversity, we hypothesized that the rhamnan backbone is conserved among *S. uberis* strains with genotype 1–4 RPS, which represents the vast majority of sequenced strains (97.5%). Additionally, by employing the anti-SKC serum which contains rhamnan-reactive antibodies, we demonstrated that the rhamnan backbone is present in both genotype 1 and 2 strains. However, as we have not analyzed genotypes 2C–2F and genotype 3 strains due to difficulties in sourcing these particular strains, this warrants further investigation of other RPS genotypes in follow-up studies. There were differences in binding of the anti-SCK serum between and within genotypes 1 and 2. Although genotype 1 strains have a single side-chain-related glycosyltransferase in the RPS biosynthesis cluster, *S. mutans* uses an unidentified glycosyltransferase outside this gene cluster to transfer Glc to the 4-position of 2-Rha [55] and genotype 1 strains could similarly have supplementary glycosyltransferases that contribute to substitutions of the backbone or side-chain. Additionally, studies on *S. mutans* have shown that the backbone is partly substituted by side-chains (i.e., not every repeat unit carries a side-chain [51, 55]). We also demonstrated that *S. uberis* 233 RPS is partly substituted by side-chains. The regulation of RPS side-chain expression remains unexplored, but variations in side-chain substitution could influence the shielding of rhamnan epitopes and consequently, antibody binding. Finally, RPS expression (backbone length and density) could be different between strains and between RPS genotypes. Based on our results we hypothesize that in genotype 4 strains, complex side-chains might shield the access of rhamnan-reactive antibodies to the RPS backbone, but compositional and structural analyses of RPS of genotype 4 strains are needed to further explore the hypothesized role of side-chain complexity in antibody binding to the backbone. Alternatively, the reduced binding of genotype 4 strains could also be a result of glycan modifications of the core rhamnose backbone (e.g., in *S. agalactiae* the backbone contains galactose, phosphate, glucitol and *N*-acetylglucosamine in addition to rhamnose [56]) or differences in side-chain substitution rates.

Interestingly, *S. pyogenes*, *S. mutans*, *S. dysgalactiae*, *S. equi*, and *S. uberis* share the same [→ 2)-α-l-Rha*p*-(1 → 3)-α-l-Rha*p*-(1 →]_n_ rhamnan as the RPS backbone [12, 16, 17, 51]. The results presented here indicate that the rhamnan backbone in the *S. uberis* RPS presents an immunogenic glycan motif in cattle. In addition, immunization of rabbits with a *S. mutans* SKC conjugate [51] induced anti-rhamnan IgG, showing that the rhamnan backbone of SKC is immunogenic as well. These observations suggest the rhamnan backbone is an immunogenic glycan motif shared across different streptococcal spp. However, we also demonstrated that depletion using isolated *S. uberis* 233 RPS resulted in effective depletion of 233 RPS-reactive IgG, but resulted in partial depletion of rhamnan-reactive IgG. Depletion of all antibodies that recognized purified 233 RPS, while antibodies that recognized synthetic rhamnan remained, suggests that not all rhamnan epitopes are accessible on the native 233 RPS. In addition, the *S. mutans* Δ*sccN* mutant (defective in the major side-chain of SCC [55]) showed much stronger binding than the parental *S. mutans* strain to anti-SKC serum. These observations suggest that immunization with a rhamnan backbone without side-chains would also induce antibodies specific for the rhamnan epitopes that are not accessible on native RPS where side-chains shield such epitopes. Immunizing with the rhamnan backbone modified with the non-immunogenic side-chains might be a novel method to induce a more targeted antibody response. Furthermore, we demonstrated that anti-SKC serum, generated by immunization of a rabbit with a SKC conjugate containing rhamnan backbone with galactose side-chains, induced antibodies that recognize the rhamnan backbones of *S. uberis* 233 RPS that has glucose side-chains (Figure 4), indicating that the rhamnan specific antibodies induced by WT RPS can access rhamnan epitopes when simple heterologous side-chains are present. However, in *S. uberis* genotype 4C strains, predicted to have the same rhamnan backbone with complex side-chains, binding of antibodies to the rhamnan epitopes was blocked. The accessibility of the rhamnan epitopes following immunization with the native type 4 RPS should be investigated to gain further insight into associations between the complexity of the immunizing RPS variant and the resultant rhamnan specificity of antibody responses and recognition of heterologous RPS variants within and between streptococcus spp.

In summary, the RPS biosynthesis gene clusters of *S. uberis* show significant genetic diversity between strains, but the genes participating in the synthesis of the rhamnan backbone are highly conserved and the vast majority of strains contain a gene encoding GroP transferase, which decorates RPS with GroP in the strain 233. The rhamnan backbone of RPS is an immunogenic glycan motif in cattle, but the binding of IgG antibodies is affected by the presence of side-chains.

## Supplementary Information

**Additional file 1. Bacteria strains used in this study.** A description of the bacteria strains used in this study.

**Additional file 2.** ***Streptococcus uberis* genomes.** A list of *Streptococcus uberis* genomes and metadata collected from publicly available database.

**Additional file 3.** **Udder and milk score.** The assessment scheme of udder and milk of the challenged cows.

**Additional file 4.** **Gating strategy for flow cytometry measurements.** Gating strategy for antibody binding assays using antigen-coated beads and bacteria. **(A–C)** Gating strategy for antigen-antibody binding assays using antigen-coated beads. **(D–F)** Gating strategy of antibody binding assays using bacteria. Single bacteria or beads were gated based on forward scatter-area (FSC-A) and side scatter-area (SSC-A). Next, the geometric mean of the Alexa Fluor 488 fluorescence intensity (GMFI) of gated populations was acquired to reflect antibody levels.

**Additional file 5.** **Phosphate quantification of *S. uberis* 233 RPS**. Phosphate quantification of purified* S. uberis* 233 RPS by malachite green assay. Purified *S. uberis* 233 RPS was acid-hydrolyzed and treated with alkaline phosphatase to release glycerol and phosphate. Rhamnose concentration was determined using a modified anthrone assay. Phosphate concentration was measured by malachite green assay and further expressed as moles per 50 moles of rhamnose.

**Additional file 6.** **NMR chemical shift prediction of the repeating unit structure of *S.*** ***uberis*** **RPS.** NMR chemical shifts of the repeating unit structure of *S. uberis* 233 RPS predicted by CASPER.

**Additional file 7.** **Supplementary material for Figure 4.** **(A) **Schematic representation of the chemical structures of the *S. uberis *233 RPS and *S. mutans *SCC variants. The glycerol phosphate modifications are not indicated in this figure. WT, wildtype. **(B)** IgG binding to *S. uberis *233 RPS before and after depletion of anti-SKC serum by different *S. uberis *genotype strains. Live bacteria were incubated with anti-SKC rabbit serum to deplete the antibodies against bacterial surface structures. Depletion of the *S. uberis *233 RPS-reactive IgG was detected by staining the *S. uberis *233 RPS-coated beads with the original (undepleted) and depleted rabbit serum and measuring antibody binding by flow cytometry. Anti-SKC serum was generated by immunizing a rabbit with SKC conjugate. *S. aureus *Newman Δ*spa*Δ*sbi *was employed as a negative control. Data from three separate bacterial inoculations are presented as mean values ± SD. **(C)** Correlation between the binding of normal rabbit serum to bacteria (Y-axis) and the respective anti-rhamnan IgG level after depletion by different bacteria at 8 × 10^5^ CFU/μL (X-axis).

**Additional file 8.** **Data for Figure** 4, **Additional file** 7**B and Figure** 5. Data for Figures 4A and B, Additional file 7B and Figure 5.

## Data Availability

The genome data of *S. uberis* 233 have been deposited at NCBI under the BioSample SAMN45088892. The other datasets used and/or analyzed during the current study are included in Additional files 1, 2, 3, 4, 5, 6, 7, 8, or are available from the corresponding author on reasonable request.
