# Supplementary material for: Discovery of glycerol phosphate and an immunogenic glycan motif in rhamnose-rich polysaccharides of Streptococcus uberis
Source: Vet Res. 2025 Jul 7;56:139. doi: 10.1186/s13567-025-01574-0 (PMC12235971; doi:10.1186/s13567-025-01574-0)
Supplement: Supplementary file 1 — Additional file 1. Bacteria strains used in this study. A description of the bacteria strains used in this study. [file 13567_2025_1574_MOESM1_ESM.pdf]

Additional file 1 Bacterial strains used in this study

Strain name	Description[a]	Reference
Streptococcus uberis		
FSL Z1-048	Subclinical mastitis isolate from the Netherlands, RPS genotype 1.	
bma	Mastitis isolate from the Netherlands, RPS genotype 1.	This study
233	Clinical mastitis isolate from New Zealand in 2003, nonencapsulated, RPS genotype 2A.	[21]
0140J	Clinical mastitis isolate from the United Kingdom, RPS genotype 2A.	
EF20	Clinical mastitis isolate from the United Kingdom, RPS genotype 2B.	
C6344	Clinical mastitis isolate from the United Kingdom, RPS genotype 2B.	[25]
C5388	Subclinical mastitis isolate from the United Kingdom, RPS genotype 4C.	[25]
C9359	Clinical mastitis isolate from the United Kingdom, RPS genotype 4C.	[25]
Streptococcus mutans		
Xc	*S. mutans* serotype c wildtype strain. Isolated from dental plaque of a healthy man.	[24]
Xc Δ*sccN*	*sccN* deletion mutant of Xc (has a nonpolar spectinomycin resistance cassette inserted in *sccN*), spc^R. The major glucose side-chains of the *S. mutans* serotype c carbohydrate (SCC) are absent (Additional file 6).	[23]
Staphylococcus aureus		
Newman Δ*spa*Δ*sbi*	A *spa* and *sbi* double knockout mutant of strain Newman.	[22]

[a] Antibiotic resistance markers: spc^R, spectinomycin.
