# Supplementary material for: Discovery of glycerol phosphate and an immunogenic glycan motif in rhamnose-rich polysaccharides of Streptococcus uberis
Source: Vet Res. 2025 Jul 7;56:139. doi: 10.1186/s13567-025-01574-0 (PMC12235971; doi:10.1186/s13567-025-01574-0)
Supplement: Supplementary file 3 — Additional file 3. Udder and milk score. The assessment scheme of udder and milk of the challenged cows. [file 13567_2025_1574_MOESM3_ESM.pdf]

Additional file 3 Udder and milk score

Throughout the experiment, udder and milk scores were assessed per challenged quarter once daily at the morning milking according to the following scheme:

Udder scores:

0 = soft pliable udder, no abnormalities

1 = slight swelling

2 = moderate swelling

3 = severe swelling

4 = other abnormalities (specify)

Milk scores:

0 = normal milk

1 = milk with some flakes or clots (< 10)

2 = milk with many flakes or clots (≥ 10)

3 = serious, watery milk with white mushy flakes

4 = yellow, thick / clotted milk

5 = other abnormalities (specify)
