# Supplementary figures and images for: Discovery of glycerol phosphate and an immunogenic glycan motif in rhamnose-rich polysaccharides of Streptococcus uberis
Source: Vet Res. 2025 Jul 7;56:139. doi: 10.1186/s13567-025-01574-0 (PMC12235971; doi:10.1186/s13567-025-01574-0)

## Slide 1
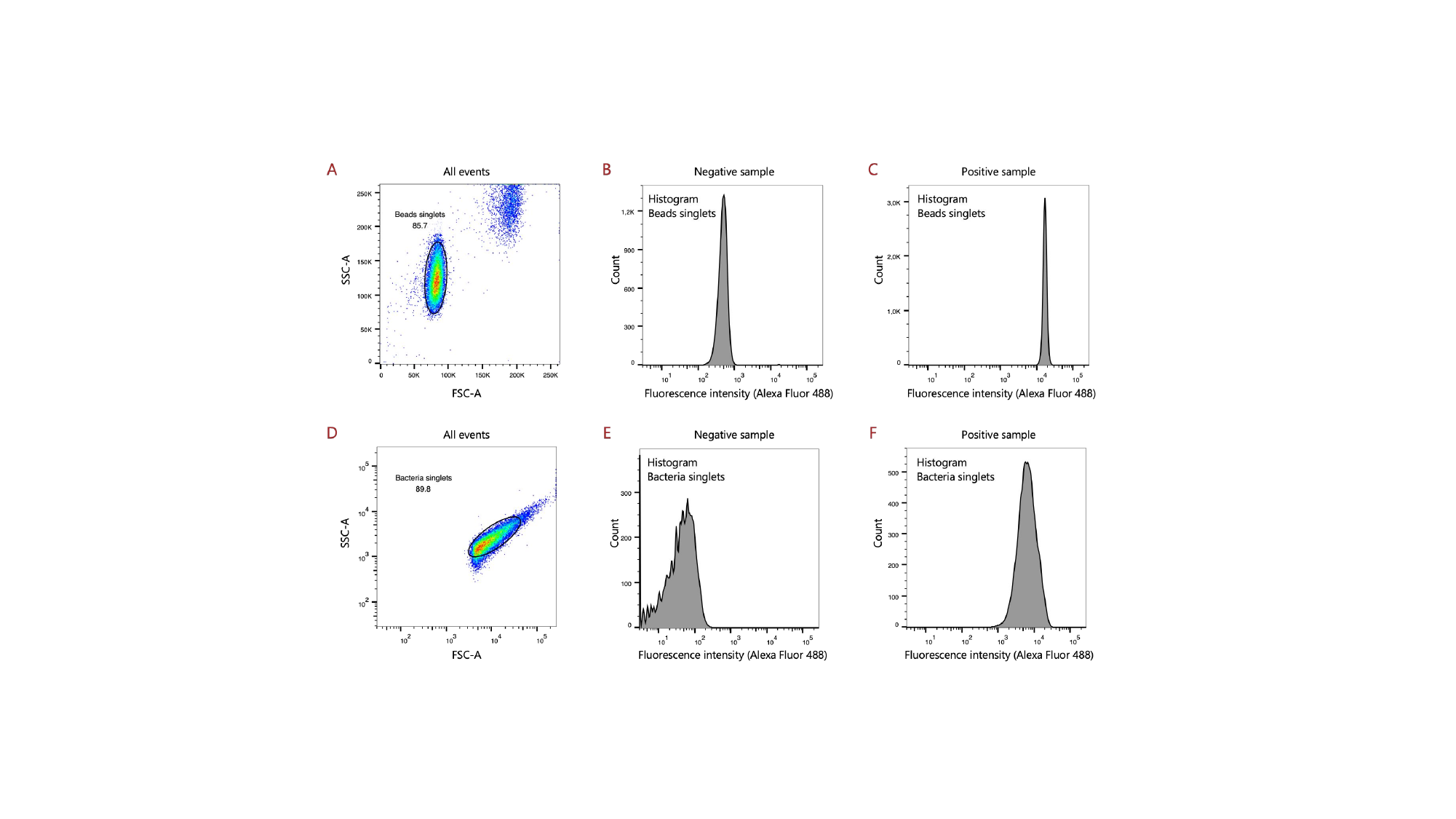

Supplement: Supplementary file 4 — Additional file 4. Gating strategy for flow cytometry measurements. Gating strategy for antibody binding assays using antigen-coated beads and bacteria. (A–C) Gating strategy for antigen-antibody binding assays using antigen-coated beads. (D–F) Gating strategy of antibody binding assays using bacteria. Single bacteria or beads were gated based on forward scatter-area (FSC-A) and side scatter-area (SSC-A). Next, the geometric mean of the Alexa Fluor 488 fluorescence intensity (GMFI) of gated populations was acquired to reflect antibody levels. [file 13567_2025_1574_MOESM4_ESM.pptx]

## Slide 1
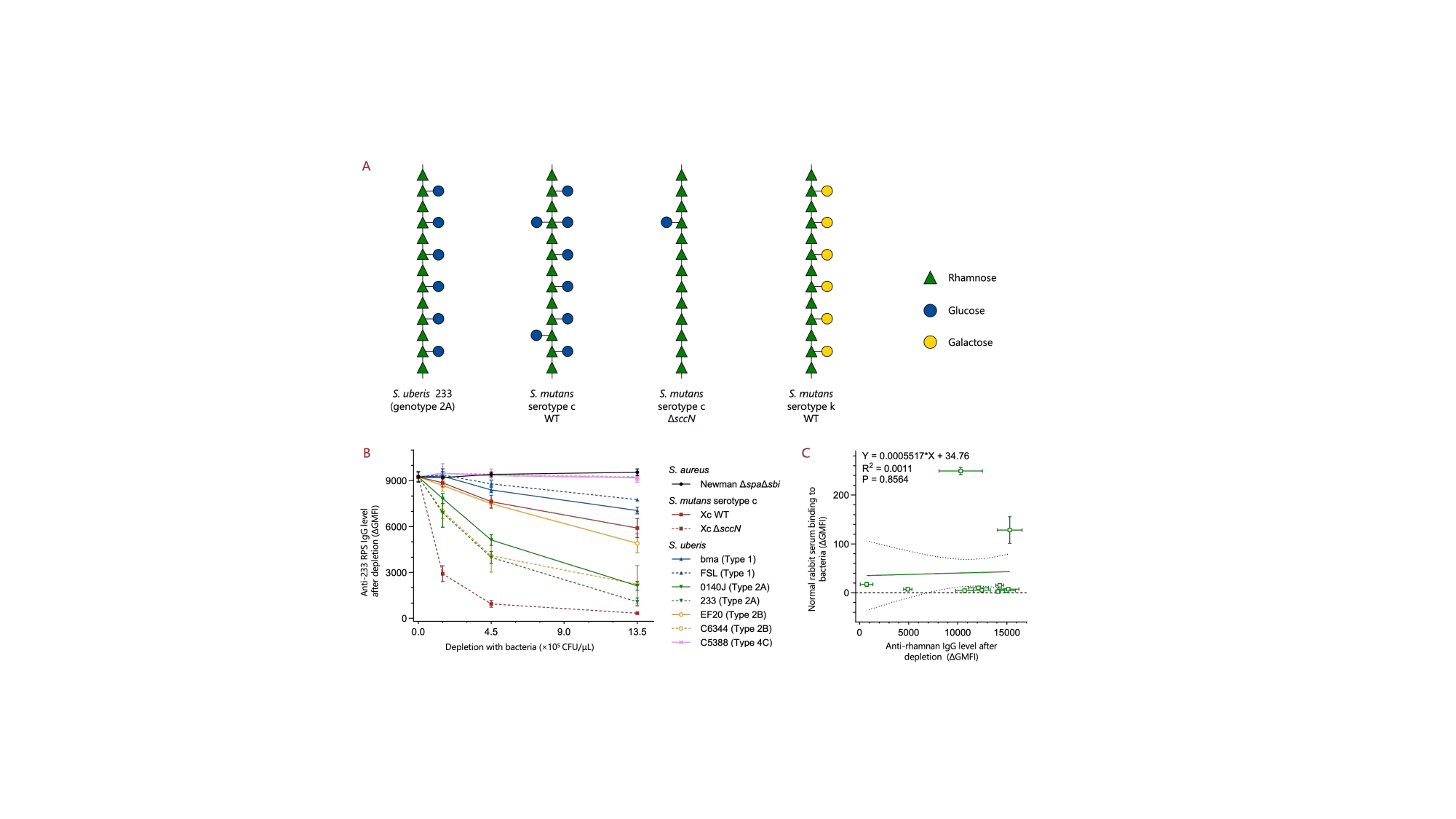

Supplement: Supplementary file 7 — Additional file 7. Supplementary material for Figure 4. (A) Schematic representation of the chemical structures of the S. uberis 233 RPS and S. mutans SCC variants. The glycerol phosphate modifications are not indicated in this figure. WT, wildtype. (B) IgG binding to S. uberis 233 RPS before and after depletion of anti-SKC serum by different S. uberis genotype strains. Live bacteria were incubated with anti-SKC rabbit serum to deplete the antibodies against bacterial surface structures. Depletion of the S. uberis 233 RPS-reactive IgG was detected by staining the S. uberis 233 RPS-coated beads with the original (undepleted) and depleted rabbit serum and measuring antibody binding by flow cytometry. Anti-SKC serum was generated by immunizing a rabbit with SKC conjugate. S. aureus Newman ΔspaΔsbi was employed as a negative control. Data from three separate bacterial inoculations are presented as mean values ± SD. (C) Correlation between the binding of normal rabbit serum to bacteria (Y-axis) and the respective anti-rhamnan IgG level after depletion by different bacteria at 8 × 10^5^ CFU/μL (X-axis). [file 13567_2025_1574_MOESM7_ESM.pptx]
