# Supplementary material for: Discovery of glycerol phosphate and an immunogenic glycan motif in rhamnose-rich polysaccharides of Streptococcus uberis
Source: Vet Res. 2025 Jul 7;56:139. doi: 10.1186/s13567-025-01574-0 (PMC12235971; doi:10.1186/s13567-025-01574-0)
Supplement: Supplementary file 5 — Additional file 5. Phosphate quantification of S. uberis 233 RPS. Phosphate quantification of purified S. uberis 233 RPS by malachite green assay. Purified S. uberis 233 RPS was acid-hydrolyzed and treated with alkaline phosphatase to release glycerol and phosphate. Rhamnose concentration was determined using a modified anthrone assay. Phosphate concentration was measured by malachite green assay and further expressed as moles per 50 moles of rhamnose. [file 13567_2025_1574_MOESM5_ESM.pdf]

Additional file 5 Phosphate quantification of purified *S. uberis* 233 RPS

Concentration	
Rhamnose	39.4 nmol/45 µL
Phosphate	1.7 nmol/45 µL
Ratio	
Phosphate per 50 Rhamnose	2.2

Purified *S. uberis* 233 RPS was acid-hydrolyzed and treated with alkaline phosphatase to release glycerol and phosphate. Rhamnose concentration was determined using a modified anthrone assay. Phosphate concentration was measured by malachite green assay and further expressed as moles per 50 moles of rhamnose.
