# Supplementary material for: Discovery of glycerol phosphate and an immunogenic glycan motif in rhamnose-rich polysaccharides of Streptococcus uberis
Source: Vet Res. 2025 Jul 7;56:139. doi: 10.1186/s13567-025-01574-0 (PMC12235971; doi:10.1186/s13567-025-01574-0)
Supplement: Supplementary file 6 — Additional file 6. NMR chemical shift prediction of the repeating unit structure of S. uberis RPS. NMR chemical shifts of the repeating unit structure of S. uberis 233 RPS predicted by CASPER. [file 13567_2025_1574_MOESM6_ESM.pdf]

**Additional file 6 NMR chemical shift prediction of the repeating unit structure of *S. uberis* RPS**

# CASPER report

## Streptococcus uberis RPS branched

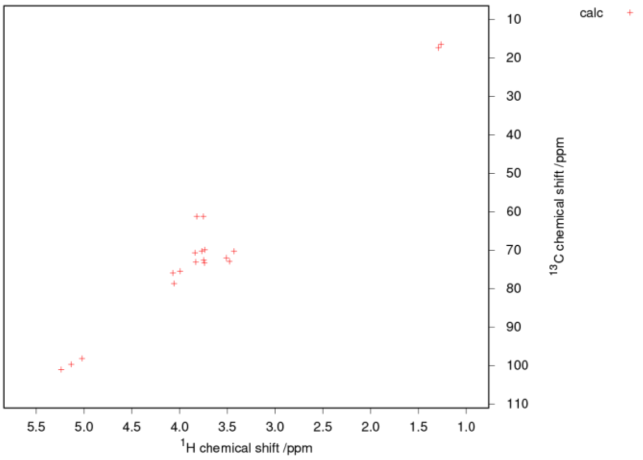


## Predicted $^{13}$C and $^{1}$H NMR chemical shifts

### Structure

→3) [α-D-Glc$^{iii}$ (1→2) ]α-L-Rha$^{ii}$ (1→2) α-L-Rha$^{i}$ (1→

| | | | | | | | |
|---|---|---|---|---|---|---|---|
| →2) α-L-Rha$^{i}$ (1→ | 1 | 2 | 3 | 4 | 5 | 6 | |
| Expected Calc. Error: 8.73 | 101.04 | 78.66 | 70.66 | 72.92 | 69.91 | 17.36 | |
| | 5.24 | 4.06 | 3.84 | 3.48 | 3.74 | 1.29 | |
| →2,3) α-L-Rha$^{ii}$ (1→ | 1 | 2 | 3 | 4 | 5 | 6 | |
| Expected Calc. Error: 9.66 | 99.70 | 75.93 | 75.47 | 72.59 | 70.25 | 16.45 | |
| | 5.13 | 4.07 | 3.99 | 3.75 | 3.77 | 1.26 | |
| α-D-Glc$^{iii}$ (1→ | 1 | 2 | 3 | 4 | 5 | 6 | 6 |
| Expected Calc. Error: 6.61 | 98.17 | 72.00 | 73.31 | 70.27 | 73.07 | 61.23 | |
| | 5.02 | 3.51 | 3.74 | 3.43 | 3.83 | 3.75 | 3.82 |

Generated 2024-11-29 10:21:12+01:00.

**Additional file 6A** NMR chemical shift prediction by CASPER of the repeating unit structure of the RPS from *S. uberis* containing an α-D-Glc*p*-(1→2)-linked side-chain residue.

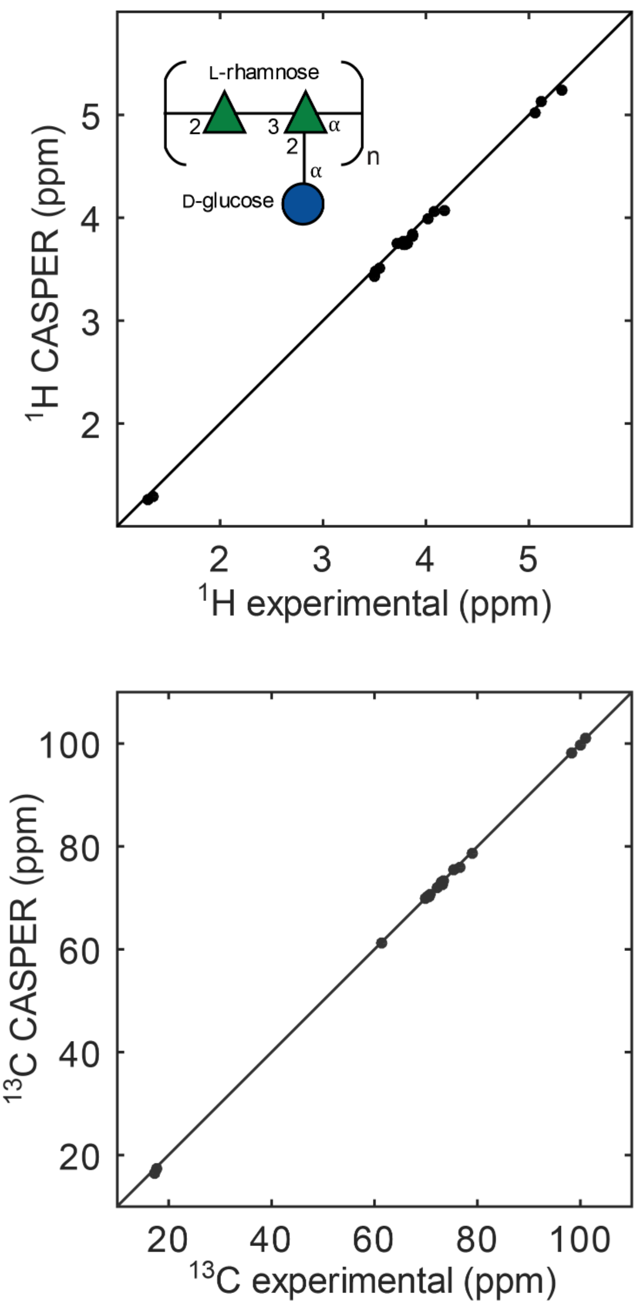


**Additional file 6B** Comparison of $^{1}$H (top) and $^{13}$C (bottom) NMR chemical shifts predicted by CASPER versus assigned by NMR experiments for the repeating unit structure (schematically inserted in the top panel) containing the α-ᴅ-Glc*p*-(1→2)-linked side-chain residue of the RPS from *S. uberis*.

# Streptococcus uberis RPS branched P-substituent

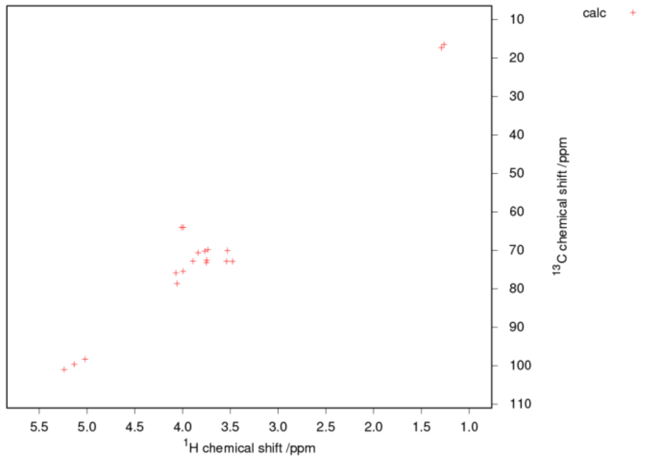


## Predicted $^{13}$C and $^{1}$H NMR chemical shifts

### Structure

→3) [α-D-Glc6P$^{iii}$ (1→2) ]α-L-Rha$^{ii}$ (1→2) α-L-Rha$^{i}$ (1→

| | | | | | | | |
|---|---|---|---|---|---|---|---|
| →2) α-L-Rha$^{i}$ (1→ | 1 | 2 | 3 | 4 | 5 | 6 | |
| Expected Calc. Error: 8.73 | 101.04 | 78.66 | 70.66 | 72.92 | 69.91 | 17.36 | |
| | 5.24 | 4.06 | 3.84 | 3.48 | 3.74 | 1.29 | |
| →2,3) α-L-Rha$^{ii}$ (1→ | 1 | 2 | 3 | 4 | 5 | 6 | |
| Expected Calc. Error: 9.66 | 99.70 | 75.93 | 75.47 | 72.59 | 70.25 | 16.45 | |
| | 5.13 | 4.07 | 3.99 | 3.75 | 3.77 | 1.26 | |
| α-D-Glc6P$^{iii}$ (1→ | 1 | 2 | 3 | 4 | 5 | 6 | 6 |
| Expected Calc. Error: 6.61 | 98.37 | 72.90 | 73.21 | 70.07 | 72.87 | 64.03 | |
| | 5.02 | 3.54 | 3.75 | 3.53 | 3.89 | 3.99 | 4.01 |

Generated 2024-11-29 10:25:08+01:00.

**Additional file 6C** NMR chemical shift prediction by CASPER of a repeating unit structure corresponding to the RPS from *S. uberis* containing an α-D-Glc*p*-(1→2)-linked side-chain residue carrying a phosphate group at O6.
